# Human host defense peptide LL-37 facilitates double-stranded RNA pro-inflammatory signaling through up-regulation of TLR3 expression in vascular smooth muscle cells

**DOI:** 10.1007/s00011-020-01340-2

**Published:** 2020-03-27

**Authors:** Sara Dahl, Samuel Cerps, Catarina Rippe, Karl Swärd, Lena Uller, Daniel Svensson, Bengt-Olof Nilsson

**Affiliations:** 1 Department of Experimental Medical Science, Lund University, BMC D12, 22184 Lund, Sweden; 2 Department of Women’s and Children’s Health, Karolinska Institutet, Box 1031, 17121 Solna, Sweden

**Keywords:** Antimicrobial peptide, Cathelicidin, Cytokines, Innate immunity, Poly I:C

## Abstract

**Objective:**

The importance of human host defense peptide LL-37 in vascular innate immunity is not understood. Here, we assess the impact of LL-37 on double-stranded RNA (dsRNA) signaling in human vascular smooth muscle cells.

**Materials and methods:**

Cellular import of LL-37 and synthetic dsRNA (poly I:C) were investigated by immunocytochemistry and fluorescence imaging. Transcript and protein expression were determined by qPCR, ELISA and Western blot. Knockdown of TLR3 was performed by siRNA.

**Results:**

LL-37 was rapidly internalized, suggesting that it has intracellular actions. Co-stimulation with poly I:C and LL-37 enhanced pro-inflammatory IL-6 and MCP-1 transcripts several fold compared to treatment with poly I:C or LL-37 alone. Poly I:C increased IL-6 and MCP-1 protein production, and this effect was potentiated by LL-37. LL-37-induced stimulation of poly I:C signaling was not associated with enhanced import of poly I:C. Treatment with poly I:C and LL-37 in combination increased expression of dsRNA receptor TLR3 compared to stimulation with poly I:C or LL-37 alone. In TLR3 knockdown cells, treatment with poly I:C and LL-37 in combination had no effect on IL-6 and MCP-1 expression, showing loss of function.

**Conclusions:**

LL-37 potentiates dsRNA-induced cytokine production through up-regulation of TLR3 expression representing a novel pro-inflammatory mechanism.

## Introduction

The human host defense peptide (HDP) LL-37 belongs to the cathelicidin family of HDPs and is well-recognized as an important part of the innate immune response [1–3]. LL-37 is produced by white blood cells and epithelial cells and formed as the pro-protein hCAP18 which is encoded by one single gene, named CAMP [4]. The hCAP18 protein is secreted and then processed extracellularly to LL-37 in a reaction catalysed by serine protease 3 and kallikrein 5 [5, 6]. LL-37 kills bacteria through permeabilization of the bacterial cell wall, and the peptide also binds and neutralizes bacterial endotoxins such as lipopolysaccharide (LPS), thereby exerting its antimicrobial activity via different mechanisms [7–10]. High concentrations of LL-37, in the micromolar range, have been demonstrated locally in the lesions of inflammatory diseases such as psoriasis, rosacea and periodontitis [11–14], and these concentrations of LL-37 have been shown to be cytotoxic for human host cells [15].

Inflammation is regarded to play an important role in the etiology and progression of atherosclerosis, and both interleukin 6 (IL-6) and monocyte chemoattractant protein 1 (MCP-1) seem to be associated with this process [16]. The atherosclerotic plaque shows high expression of LL-37 both on the transcript and protein levels in comparison to normal arterial tissue [17]. In the plaque, LL-37 immunoreactivity is mainly observed in macrophages [17]. The atherosclerotic plaques also contain smooth muscle cells which indeed represent a major cell type of the plaque, and LL-37 produced by the macrophages may affect the functional properties of the adjacent smooth muscle cells. Although it is suggested that LL-37 can modulate the innate immune response in atherosclerosis, the LL-37-induced effects on vascular innate immune responses and the underlying mechanisms of action are not understood [17]. Interestingly, LL-37 has been shown to sense and recognize microbial DNA, facilitate oligonucleotide delivery and promote import of nucleic acids by human host cells [18, 19]. In human airway epithelial cells, LL-37 has been demonstrated to bind double-stranded RNA (dsRNA), and this complex is thought to enter endosomes [20]. Although dsRNA is clearly associated with viruses and viral infections it is also well documented that dsRNA is endogenously synthesized by host cells [21]. Upon endosomal acidification, dsRNA is released from LL-37 and the dsRNA then activates toll-like receptor (TLR) 3 [20]. TLR3 is widely recognized to display intracellular localization and to recognize dsRNA in the endolysosomal compartments [22–24]. Thus, there are reports describing interactions between LL-37 and dsRNA signaling, but the mechanisms behind LL-37-induced modulation of these processes are poorly understood.

The aim of the present study was to assess the effects of LL-37 on dsRNA signaling in human vascular smooth muscle cells and investigate underlying mechanisms. We demonstrate that LL-37 potentiates dsRNA-induced production of the pro-inflammatory cytokines IL-6 and MCP-1 via up-regulation of the dsRNA receptor TLR3, offering a novel mechanism by which LL-37 may modulate vascular innate immunity.

## Materials and methods

### Cells and cell culture

Human coronary artery smooth muscle cells (hCASMCs) were purchased from Thermo Fisher Scientific (Waltham, MA, USA) and cultured in Medium 231 (Thermo Fisher Scientific), supplemented with smooth muscle growth supplement (Thermo Fisher Scientific) and antibiotics (penicillin 50 U/ml, streptomycin 50 µg/ml, Biochrom GmbH, Berlin, Germany). The cells were grown in a water-jacked cell incubator at 37 °C with 5% CO_2_ in air, and medium was exchanged every second day. Cells were trypsinized upon reaching confluence, counted using a LUNA automated cell counter (Logos Biosystems, Annandale, VA, USA) and re-seeded. Cells were used for experiments in passages 3–10.

### Determination of cell morphology, cell number and cell viability

Cell morphology was assessed using a phase-contrast microscope (Olympus CKX41, Olympus Europa GmbH, Hamburg, Germany) equipped with an Olypmus SC50 5-megapixel digital camera (Olympus), and the number of cells was determined using a LUNA automated cell counter (Logos Biosystems). Cell viability was assessed by the thiazolyl blue tetrazolium bromide (MTT) assay as previously described by Svensson et al. [15]. Briefly, cells were incubated with MTT solution (0.5 mg/ml, Sigma Aldrich, St Louis, MO, USA) for 1 h at 37 °C in the cell incubator. After incubation, the supernatant was discarded and the formazan product dissolved in DMSO. The absorbance was read at 540 nm in a Multiskan GO Microplate Spectrophotometer (Thermo Fisher Scientific).

### Immunocytochemistry

Cells were cultured on glass coverslips and stimulated with or without 1 µM LL-37 for 4 h at 37 °C. The cells were washed in phosphate-buffered saline (PBS) and then fixed with 4% paraformaldehyde for 10 min, followed by permeabilization with 0.1% Triton-X for 10 min. Non-specific binding sites were blocked with 2% bovine serum albumin (BSA) in PBS for 1 h, and the cells were then incubated for 2 h with a monoclonal mouse primary LL-37 antibody [25], diluted 1:400 in 2% BSA in PBS. After incubation with the primary antibody, cells were washed and incubated for 1 h with a secondary anti-mouse Alexa Flour 488 antibody (Thermo Fisher Scientific). The coverslips were mounted with Fluoroshield DAPI (Sigma Aldrich) on glass slides before being analysed in a fluorescence microscope (Olympus BX60, Olympus), coupled to an Olympus DP72 digital camera (Olympus). DAPI was included as a nuclear marker. Immunoreactivity for LL-37 was not observed after omission of the primary antibody.

### Fluorescence imaging of poly I:C import

Cells were cultured on glass coverslips and stimulated with or without fluorescent rhodamine-tagged polyinosine-polycytidylic acid (poly I:C, 1 µg/ml), purchased from InvivoGen (San Diego, CA, USA), in the absence or presence of LL-37 (1 µM) for 24 h at 37 °C. After stimulation, cells were washed in PBS and fixed with 4% paraformaldehyde for 10 min. The coverslips were mounted with Fluoroshield DAPI (Sigma-Aldrich) on glass slides before being analysed using an Olympus BX60 fluorescence microscope. DAPI was included as a nuclear marker.

### Transfection with TLR3 siRNA

TLR3 siRNA (#AM16708) and scrambled negative-control (NC) siRNA, both from Ambion Thermo Fisher Scientific, were transfected into hCASMCs using Oligofectamine transfection reagent (Thermo Fisher Scientific) as described by Akbarshahi et al. [26]. Briefly, the cells were treated with 100 nM of either TLR3 siRNA or NC for 24 h in Opti-MEM medium (Thermo Fisher Scientific) followed by incubation with TLR3 siRNA or NC, at 50 nM each, in a mixture (1:1) of Opti-MEM medium and Medium 231 for another 48 h. The cells were stimulated with or without poly I:C (10 µg/ml) and LL-37 (1 µM) in combination for the last 24 h of the 72 h transfection period. Knockdown of TLR3 was confirmed using quantitative real-time RT-PCR analysis.

### Quantitative real-time RT-PCR

Total RNA was extracted and purified from hCASMCs with a miRNeasy kit (Qiagen, Venlo The Netherlands), using the QIAcube (Qiagen) as recommended by the manufacturer. Briefly, cells were lysed in QIAzol and the RNA percipitated using chloroform. The RNA was washed using miRNeasy mini spin columns and eluated in RNease-free water. The concentration and quality of RNA were determined using a NanoDrop 2000C spectrophotometer (Thermo Fisher Scientific). Transcript levels were analysed on a Step One Plus real-time thermal cycler (Applied Biosystems, Waltham, MA, USA) in an one-step quantitative real-time RT-PCR measurement, using the QuantiFast SYBR Green RT-PCR kit (Qiagen) as described previously [15]. The samples were analysed in duplicate and GAPDH was included as a housekeeping gene. Gene expression was calculated using the delta CT method [27]. Primers for MCP-1 (Hs_CCL2_1_SG), IL-6 (Hs_IL6_1_SG), TLR3 (Hs_TLR3_1_SG) and GAPDH (Hs_GAPDH_2_SG) were purchased from Qiagen.

### ELISA

An enzyme-linked immunosorbent assay (ELISA) was used to determine IL-6 and MCP-1 protein levels. The cells were collected in ice-cold PBS and sonicated for 2 × 10 s on ice. The homogenate was centrifuged at 1700×*g* for 5 min at 4 °C and the supernatants collected. The levels of IL-6 and MCP-1 were measured in the supernatants using Quantikine ELISA Human IL-6 Immunoassay (R&D systems, #D6050, Minneapolis, MN, USA) and Quantikine ELISA Human MCP-1 Immunoassay (R&D systems, #DCP00), according to manufacturer’s protocol. IL-6 and MCP-1 concentrations were normalized to the amount of total protein in each sample. The total protein concentration was determined using the Bio-Rad DC protein assay (Bio-Rad, Hercules, CA, USA), according to manufacturer´s instructions.

### Western blot

Western blot analysis was performed as described previously with minor modifications [15]. Briefly, proteins were separated by SDS/PAGE on Criterion TGX 4–15% precast gels (Bio-Rad) and transferred to nitrocellulose membranes using a Trans-Blot Turbo Transfer system (Bio-Rad). The membranes were blocked in 0.5% casein in Tris-buffered saline and incubated overnight with a rabbit monoclonal TLR3 antibody (Cell Signaling, #6961S, Danvers, MA, USA) diluted 1:1000 and a mouse monoclonal GAPDH antibody (clone 6C5 Merck Millipore, #MAB374, Burlington, MA, USA) diluted 1:3000. Immunoreactive bands were visualized using horseradish peroxidase-conjugated secondary anti-rabbit and anti-mouse antibodies and SuperSignal West Femto chemiluminescence reagent (Thermo Fisher Scientific). The TLR3 band was analysed by densitometric scanning and normalized to GAPDH using a LI-COR Odyssey Fc instrument (LI-COR Biosciences).

### Agents

Synthetic LL-37 was purchased from Bachem (Bubendorf, Switzerland) and disolved in DMSO. Polyinosine-polycytidylic acid (poly I:C, InvivoGen, high molecular weight), fluorescent rhodamine-tagged poly I:C (InvivoGen) and LPS (*Escherichia coli* 0111:B4, Sigma-Aldrich) were dissolved in PBS. Vehicle was included as appropriate.

### Statistics

The data were analyzed using GraphPad Prism7 (GraphPad Software) and presented as means ± SEM. Each experiment was repeated at least two times, where each culture well represents one biological replicate (*n* = 1). The n-values are displayed in the figure legends. Statistical significance was calculated using one-way ANOVA followed by Dunnett’s or Tukey’s multiple comparison tests as appropriate.

## Results

### Low concentrations of LL-37 have no effect on cell viability

**Fig. 1:**
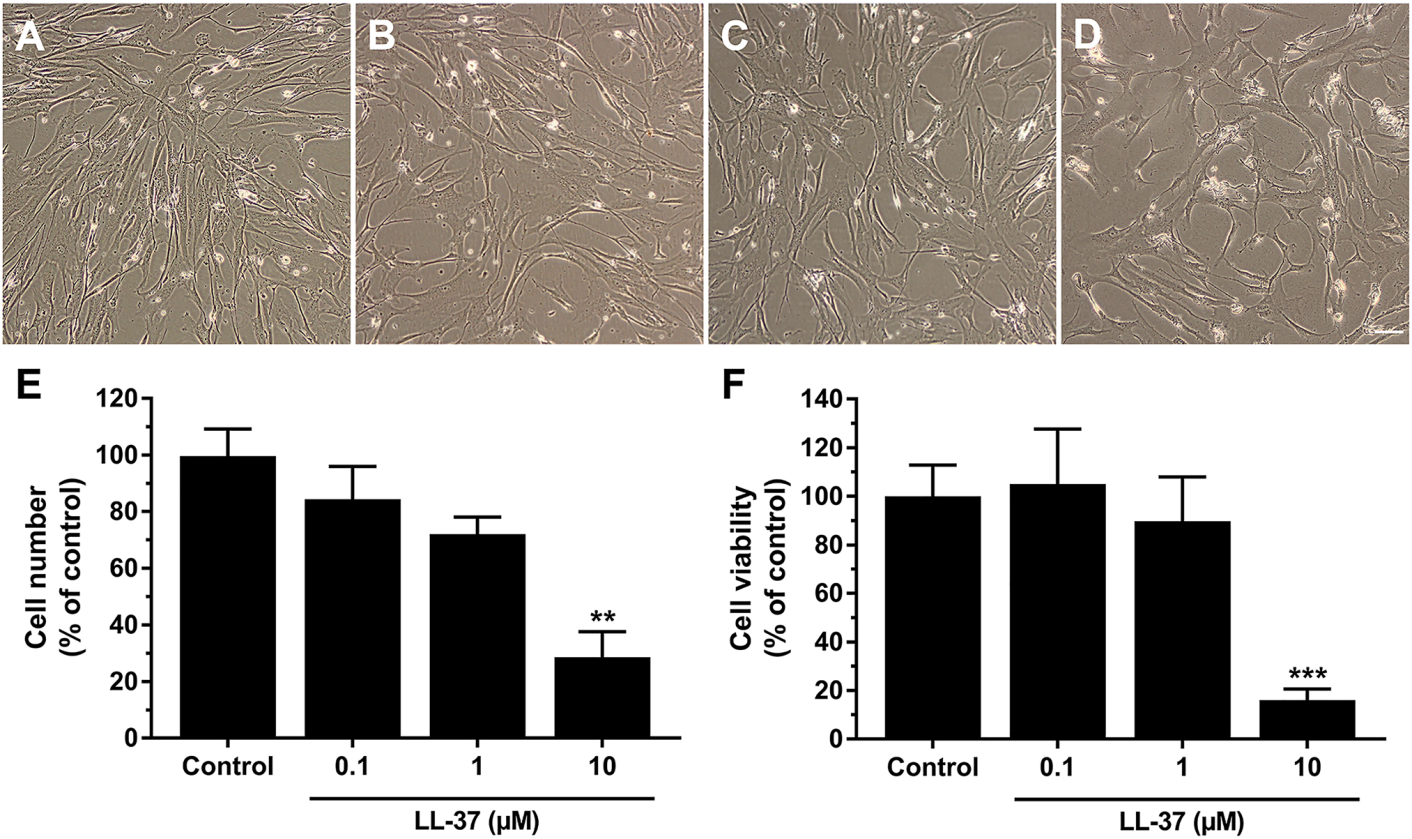
LL-37 reduces hCASMC cell number and viability. Phase-contrast microscopic pictures showing morphology of control cells (**a**) and cells treated with 0.1 (**b**), 1 (**c**) and 10 (**d**) µM LL-37 for 72 h. Bar in **d** represents 50 µm and applies to panels **a**–**d**. Cell morphology was assessed by phase-contrast microscopy using an Olympus CKX41 microscope (Olympus). **e** Cell counting shows that treatment with 10 µM LL-37 for 72 h reduces cell number by about 75%. **f** High (10 µM), but not low (0.1 and 1 µM), concentrations of LL-37 reduce cell viability. The cells were treated with or without LL-37 for 4 h and cell viability assessed by the MTT method. Values are presented as mean ± SEM of 3–4 observations in each group. ** and *** represent *P* < 0.01 and *P* < 0.001, respectively, vs. control

LL-37 has been reported to reduce human host cell viability [15], and, therefore, we started by examining the effects of synthetic LL-37 on hCASMC cell number, morphology and cell viability. In the first set of experiments, we assessed effects of LL-37 on hCASMC cell number and morphology. Treatment with 10 µM LL-37 for 72 h reduced the number of cells by about 75%, whereas lower concentrations of LL-37 (0.1 and 1 µM) had no effect (Fig. 1a–e). As noted by phase-contrast microscopic analysis, LL-37 (0.1–10 µM) had no appreciable effect on cell morphology (Fig. 1a–d). Next, we assessed the effects of LL-37 on hCASMC viability using the MTT method. Treatment with 10 µM LL-37 for 4 h reduced cell viability by 80%, but lower concentrations of LL-37 (0.1 and 1 µM) had no effect (Fig. 1f). Because 1 µM LL-37 had no effect on either cell number or cell viability, we decided to use this concentration of the peptide throughout the study.

### LL-37 is internalized by hCASMCs

**Fig. 2:**
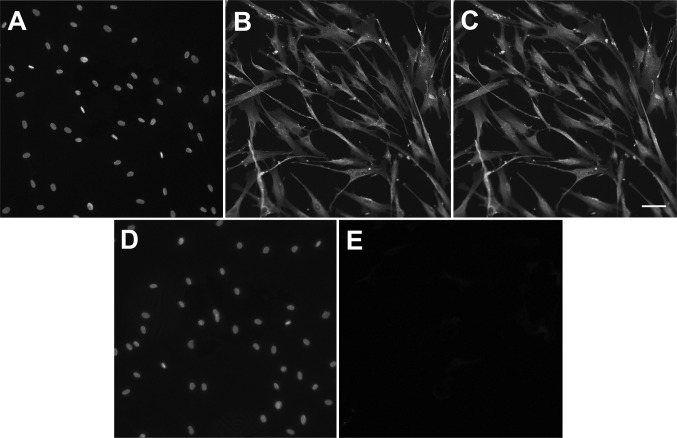
LL-37 is internalized by hCASMCs. **a**–**e** Cells treated with LL-37 (1 µM) for 4 h possess cytoplasmic immunoreactivity for LL-37 (green colour, **a**–**c**). Overlay is shown in **c**. No immunoreactivity for LL-37 was observed in untreated, control cells (**d**, **e**). Nuclei were stained with DAPI (blue colour). The immunoreactivity for LL-37 and DAPI staining were evaluated using an Olympus BX60 fluorescence microscope (Olympus). Bar in **c** represents 50 µm for all panels

To assess if exogenous LL-37 is imported by hCASMCs, we treated the cells with synthesized LL-37 and then performed immunocytochemical analysis for LL-37 immunoreactivity. In cells treated with LL-37 (1 µM) for 4 h, cytoplasmic immunoreactivity for LL-37 was observed in all cells, whereas untreated control cells expressed no immunoreactivity (Fig. 2a–e). No or very weak LL-37 immunoreactivity was seen in nuclei of cells treated with LL-37 (Fig. 2a–e). In negative control cells where the primary LL-37 antibody was omitted, no immunoreactivity was observed (data not shown). Thus, we can conclude that LL-37 is rapidly internalized by the hCASMCs.

### LL-37 promotes poly I:C-induced expression of pro-inflammatory IL-6 and MCP-1 in hCASMCs

**Fig. 3:**
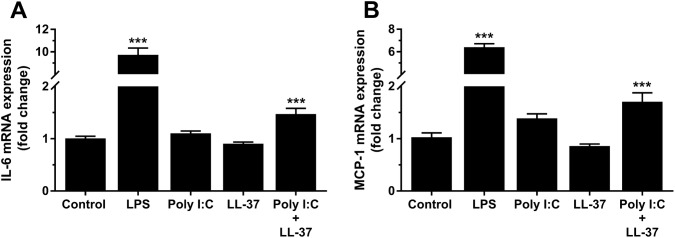
Poly I:C promotes pro-inflammatory IL-6 and MCP-1 hCASMC mRNA expression in the presence of LL-37. Cells were stimulated with poly I:C (10 µg/ml) for 24 h in the presence or absence of LL-37 (1 µM) and transcript expression of IL-6 (**a**) and MCP-1 (**b**) assessed by quantitative real-time RT-PCR. LPS (0.1 µg/ml) was included as positive control and enhanced both IL-6 and MCP-1 mRNA levels several fold (**a**, **b**). Values are presented as mean ± SEM of 8 observations in each group. *** represents *P* < 0.001 vs. control

**Fig. 4:**
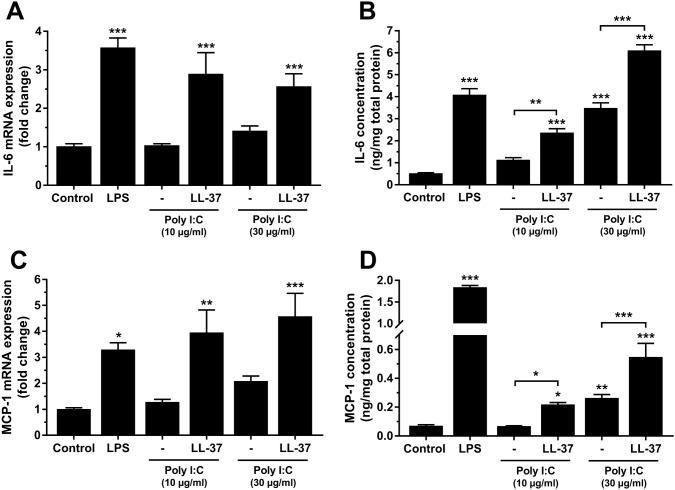
LL-37 potentiates poly I:C-induced IL-6 and MCP-1 transcript and protein production in hCASMCs. Cells were stimulated with poly IC (10 and 30 µg/ml) for 24 h in the presence or absence of LL-37 (1 µM) and transcript and protein expression of IL-6 (**a**, **b**) and MCP-1 (**c**, **d**) were assessed by quantitative real-time RT-PCR and ELISA, respectively. LPS (0.1 µg/ml) was included as positive control. Values are presented as mean ± SEM of 4–8 observations in each group. *, ** and *** represent *P* < 0.05, *P* < 0.01 and *P* < 0.001, respectively, vs. control. For comparisons indicated by horizontal bars, *, ** and *** represent *P* < 0.05, *P* < 0.01 and *P* < 0.001, respectively

In the next experiments, we investigated effects of synthetic dsRNA (poly I:C) on the transcript expression of IL-6 and MCP-1 in hCASMCs treated either with or without LL-37. Stimulation with LPS (0.1 µg/ml) for 24 h, included as a positive control, increased IL-6 and MCP-1 expression by about 10 and 7 times, respectively (Fig. 3a, b). Treatment with poly I:C (10 µg/ml) for 24 h enhanced both IL-6 and MCP-1 mRNA expression in the presence but not in the absence of LL-37 (1 µM), showing that LL-37 facilitates the poly I:C-induced pro-inflammatory cytokine expression (Fig. 3a, b). Incubation with LL-37 alone had no effect on either IL-6 or MCP-1 expression (Fig. 3a, b). Next, we investigated the effects of different concentrations (10 and 30 µg/ml) of poly I:C on IL-6 and MCP-1 mRNA expression and protein production in the presence or absence of LL-37. Treatment with poly I:C alone (10 or 30 µg/ml) for 24 h had no statistically significant effect on either IL-6 or MCP-1 mRNA expression, although the high concentration of poly I:C (30 µg/ml) tended to enhance both IL-6 and MCP-1 transcript levels on its own (Fig. 4a, c). Stimulation with poly I:C (10 and 30 µg/ml) and LL-37 (1 µM) in combination increased the IL-6 transcript about three times and the MCP-1 transcript about four times (Fig. 4a, c). Assessment of IL-6 and MCP-1 protein production by ELISA showed that stimulation with the low concentration of poly I:C (10 µg/ml) for 24 h had no effect on either IL-6 or MCP-1 protein concentration, whereas the high concentration (30 µg/ml) increased both IL-6 and MCP-1 concentration severalfold on its own (Fig. 4b, d). Co-stimulation with poly I:C (10 and 30 µg/ml) and 1 µM LL-37 enhanced IL-6 and MCP-1 protein concentrations by about two times compared to stimulation with each concentration of poly I:C alone, showing that LL-37 strongly potentiates the effect of poly I:C (Fig. 4b, d). Overall, these results show that LL-37 potentiates both poly I:C-induced IL-6 and MCP-1 expression.

### LL-37 has no effect on import of poly I:C by hCASMCs

**Fig. 5:**
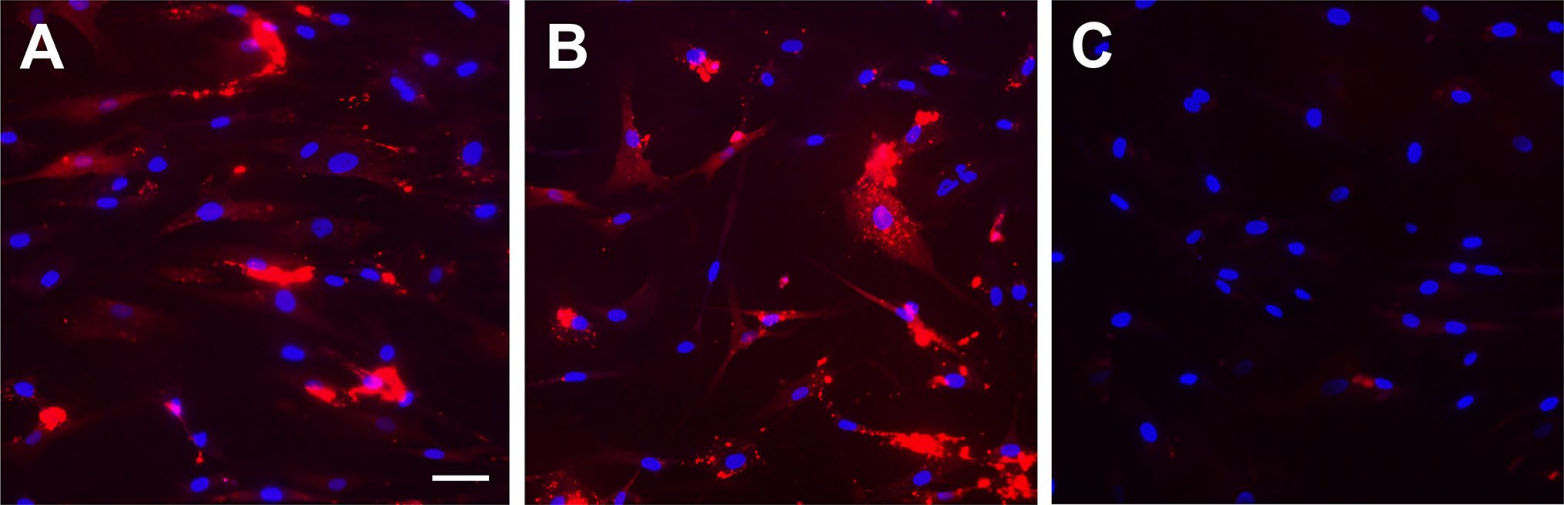
Poly I:C is internalized by hCASMCs. Both cells treated with fluorescent rhodamine-tagged poly I:C (1 µg/ml) alone (**a**) and cells incubated with rhodamine-tagged poly I:C (1 µg/ml) and 1 µM LL-37 in combination (**b**) for 24 h show intracellular fluorescence (red colour). Untreated, control cells displayed no fluorescence (**c**). Nuclei were stained with DAPI (blue colour). Fluorescence and DAPI staining were analysed using an Olympus BX60 fluorescence microscope (Olympus). Bar in **a** represents 50 µm for all panels

LL-37-induced potentiation of the poly I:C effect may be due to LL-37-evoked stimulation of cellular poly I:C import. Therefore, we assessed internalization of fluorescent rhodamine-tagged poly I:C by hCASMCs in the presence or absence of LL-37 (Fig. 5a–c). An intracellular and cytosolic fluorescence signal, distinctly stronger than that for untreated control cells, was observed in cells incubated with rhodamine-tagged poly I:C (1 µg/ml) for 24 h (Fig. 5a). Co-stimulation with rhodamine-tagged poly I:C (1 µg/ml) and LL-37 (1 µM) for 24 h did not enhance import of poly I:C compared to cells incubated with fluorescent poly I:C alone (Fig. 5a, b). No or very weak fluorescence signal was detected in untreated control cells (Fig. 5c). Hence, we can conclude that LL-37 does not promote cellular internalization of poly I:C.

### LL-37 up-regulates hCASMC TLR3 expression in the presence of poly I:C

**Fig. 6:**
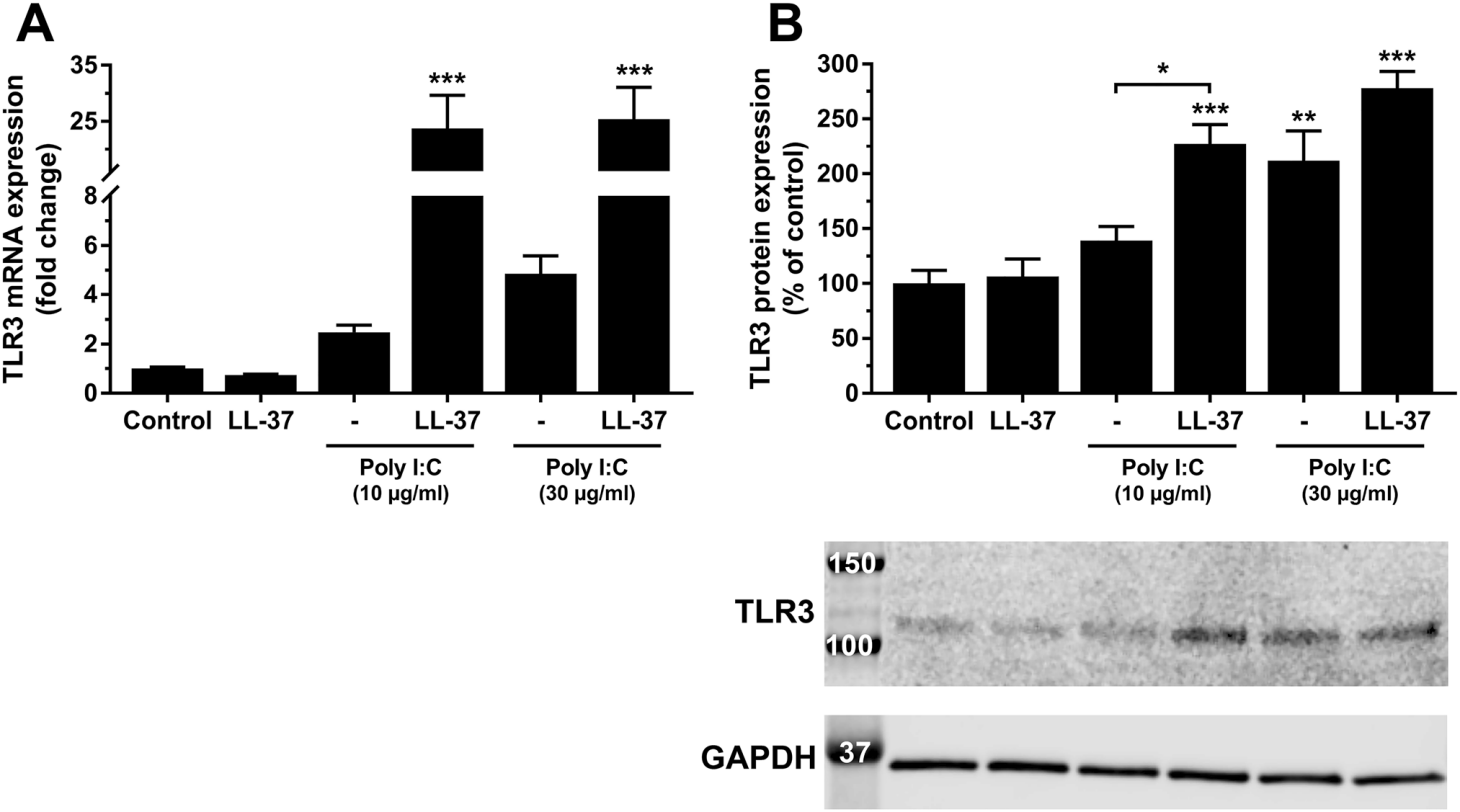
Poly I:C enhances hCASMC TLR3 expression in the presence of LL-37. Cells were treated with either poly I:C (10 and 30 µg/ml) or LL-37 (1 µM) alone or the two in combination for 24 h and TLR3 mRNA (**a**) and protein (**b**) levels determined by quantitative real-time RT-PCR and Western blot, respectively. For Western blot, the TLR3 immunoreactive band was observed at the expected molecular weight of 115–130 kDa and normalized to GAPDH serving as internal control. Values are presented as mean ± SEM of 6–8 observations in each group. ** and *** represent *P* < 0.01 and *P* < 0.001, respectively, vs. control. For comparison indicated by the horizontal bar, * represents *P* < 0.05

Since LL-37 had no effect on the internalization of poly I:C, we looked for down-stream effects of LL-37 on poly I:C/TLR3 signaling. We hypothesized that LL-37 may up-regulate TLR3 expression in the presence of poly I:C and assessed this possibility by measuring TLR3 transcript expression in cells stimulated with either poly I:C or LL-37 alone or cells treated with poly I:C and LL-37 in combination. Stimulation with poly I:C (10 and 30 µg/ml) alone for 24 h tended to increase TLR3 mRNA expression, but this effect did not reach statistical significance, whereas stimulation with poly I:C (10 or 30 µg/ml) and LL-37 (1 µM) in combination elevated the TLR3 transcript by about 25 times (Fig. 6a). Treatment with LL-37 (1 µM) alone had no effect on TLR3 mRNA levels (Fig. 6a). Next, we assessed the effects of treatment with poly I:C or LL-37 alone or the two in combination on TLR3 protein expression using Western blot. Stimulation with LL-37 (1 µM) or 10 µg/ml poly I:C alone for 24 h had no effect on TLR3 protein expression, whereas treatment with 30 µg/ml poly I:C alone increased TLR3 protein levels by about two times (Fig. 6b). Stimulation with poly I:C (10 µg/ml) and LL-37 in combination increased TLR3 protein by about 70% compared to treatment with poly I:C alone, while co-stimulation with 30 µg/ml poly I:C and LL-37 had no additive effect compared to stimulation with poly I:C alone (Fig. 6b). Thus, we show that stimulation with poly I:C and LL-37 in combination enhances TLR3 expression.

### Knockdown of TLR3 gene activity abolishes poly I:C/LL-37-induced stimulation of IL-6 and MCP-1 expression in hCASMCs

**Fig. 7:**
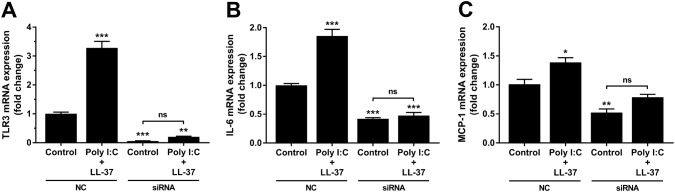
TLR3 knockdown hCASMCs show loss of function. **a**–**c** Cells were treated with either TLR3 siRNA or negative-control (NC) scrambled construct for 72 h and stimulated with or without poly I:C (10 µg/ml) and LL-37 (1 µM) in combination for the last 24 h of the transfection period. Knockdown of TLR3 was confirmed by quantitative real-time RT-PCR (**a**). Stimulation with poly I:C (10 µg/ml) and LL-37 (1 µM) in combination increased IL-6 and MCP-1 transcripts in NC-treated but not in TLR3 siRNA-treated cells (**b**, **c**). Values are presented as mean ± SEM of four observations in each group. *, ** and *** represent *P* < 0.05, *P* < 0.01 and *P* < 0.001, respectively vs. NC control. For comparisons indicated by horizontal bars, ns represents non-significant

In the next experiments, we used TLR3 siRNA to study loss of function after down-regulation of TLR3 gene activity. Treatment with TLR3 siRNA for 72 h reduced hCASMC TLR3 transcript level by about 95% compared to cells treated with negative-control (NC) scrambled construct, ensuring successful knockdown of TLR3 gene activity (Fig. 7a). Since treatment with poly I:C and LL-37 in combination up-regulates TLR3 expression, we also assessed the effects of TLR3 siRNA in these cells. In NC-treated cells, stimulation with poly I:C (10 µg/ml) and LL-37 (1 µM) in combination for the last 24 h of the 72 h transfection period increased TLR3 mRNA expression about three times compared to NC-treated control cells, whereas treatment with TLR3 siRNA completely blunted the stimulatory effect of poly I:C and LL-37 on TLR3 expression and reduced TLR3 expression by about 80% compared to NC-treated control cells (Fig. 7a). Thus, we observed successful knockdown of TLR3 both in control cells and in cells co-stimulated with poly I:C and LL-37. Stimulation with poly I:C (10 µg/ml) and LL-37 (1 µM) in combination for the past 24 h of the transfection period increased IL-6 and MCP-1 mRNA expression by about two times and 50%, respectively, in cells treated with NC, whereas it had no effect in cells treated with siRNA for TLR3 (Fig. 7b, c). Hence, cells treated with TLR3 siRNA showed loss of function.

## Discussion

In the present study, we show that LL-37 potentiates dsRNA-induced stimulation of pro-inflammatory IL-6 and MCP-1 production in hCASMCs through a mechanism that involves up-regulation of the dsRNA receptor TLR3. The functional importance of this mechanism was demonstrated using TLR3 siRNA and loss of function analysis. Importantly, IL-6 and MCP-1 are thought to play essential roles in vascular inflammation and in the initiation and progression of atherosclerosis [16]. We suggest that LL-37 may enhance the vascular innate immune response through up-regulation of TLR3 in the presence of dsRNA providing a novel LL-37-induced pro-inflammatory mechanism of action.

Here, we show that high (> 4 µM), but not low, concentrations of LL-37 reduce hCASMC viability. Indeed, high concentrations of LL-37 have been demonstrated to be cytotoxic in other human host cell types, and this effect seems to be inversely correlated to cell type specific expression of the human protein p33, also named globular C1q receptor [15]. Notably, we used 1 µM of LL-37 throughout this study, representing a concentration of the peptide which possesses no negative impact on cell viability. Interestingly, LL-37-evoked release of nucleic acids has been reported in human mast cells, indicating that LL-37 may induce release of endogenous dsRNA, which then can promote a vascular inflammation response in the presence of LL-37 through up-regulation of TLR3 expression as demonstrated in the present study, a paracrine mechanism which can be of physiological/pathophysiological in vivo importance [28]. Interestingly, high expression of LL-37 has been reported in atherosclerotic lesions vs. healthy vascular tissue [17], and furthermore, Lundberg et al. [29] have reported that deleting TLR3 in immune cells attenuates both aortic inflammation and atherosclerosis in mice, suggesting that LL-37-induced potentiation of TLR3 signaling indeed can be of pathophysiological importance.

In the present study, we demonstrate that LL-37 is rapidly internalized by hCASMCs indicating that LL-37 shows intracellular mechanisms of action. However, LL-37 does not enhance import of dsRNA as demonstrated in the present study using fluorescent poly I:C, suggesting that LL-37 facilitates dsRNA-induced signaling and pro-inflammatory responses via a mechanism down-stream of the plasma membrane. We have previously reported that LL-37 is imported through clathrin-mediated endocytosis, but this process only seems to have minor importance for LL-37-induced human host cell cytotoxicity, indicating that LL-37-evoked cytotoxicity is dependent on internalization of LL-37 through other pathways than clathrin-mediated endocytosis [30]. It seems reasonable to conclude that import of LL-37 by human host cells may cause both modulation of the innate immune response as reported in the present study and cytotoxicity.

We demonstrate that dsRNA can induce production of pro-inflammatory cytokines on its own and is internalized by the hCASMCs also in the absence of LL-37, suggesting that internalization of dsRNA and activation of its intracellular TLR3 receptor is not critically dependent on LL-37, although the peptide strongly potentiates the dsRNA-induced vascular inflammation response through up-regulation of TLR3 expression. Thus, we conclude that LL-37 facilitates dsRNA-induced pro-inflammatory responses via this mechanism. LL-37 has recently been reported to enhance dsRNA/TLR3 signaling in human airway epithelial cells, although the underlying mechanisms have not been fully clarified [20, 31]. Singh et al. [20] provide evidence that LL-37 binds dsRNA forming a complex, and that dsRNA is released from LL-37 in response to acidification of the endosomes. This process is thought to elevate the amount of free dsRNA that is available for binding and activation of TLR3. Hence, Singh et al. [20] suggest that lowering endosomal pH can increase intracellular levels of free and biologically active dsRNA. Here, we show that LL-37, in the presence of dsRNA, enhances TLR3 expression, and that this mechanism is necessary for LL-37-induced potentiation of dsRNA-evoked production of pro-inflammatory IL-6 and MCP-1. Thus, LL-37 may modulate the innate immune system both via increasing the intracellular bioavailability of dsRNA as shown by Singh et al. [20] and through up-regulation of TLR3, occurring in the presence of dsRNA, as demonstrated in the present study.
